# Biliary cannulation of a peridiverticular papilla using multiloop traction in a patient with Roux-en-Y anatomy

**DOI:** 10.1055/a-2440-6201

**Published:** 2024-11-22

**Authors:** Kumi Itami, Kosuke Iwano, Shujiro Yazumi

**Affiliations:** 1 566610 Gastroenterology and Hepatology, Kitano Hospital Medical Research Institute, Osaka, Japan

Post-surgical altered anatomy is a hurdle for biliary cannulation during endoscopic retrograde cholangiopancreatography (ERCP) 1 . The innovative multiloop traction method has been described for difficult biliary cannulation because of periampullary diverticula 2 3 . We report successful biliary cannulation using the multiloop method with double-balloon ERCP for a peridiverticular papilla.

A 76-year-old man with a history of distal gastrectomy with Roux-en-Y reconstruction was admitted to our hospital with epigastric pain and was diagnosed with a common bile duct stone. Double-balloon ERCP was attempted, but the bile duct opening was not identified because of the peridiverticular papilla. Therefore, the multiloop technique was applied ( Fig. 1 , Video 1 ) to pull the papilla out of the diverticulum and reveal the bile duct opening.

**Fig. 1:**
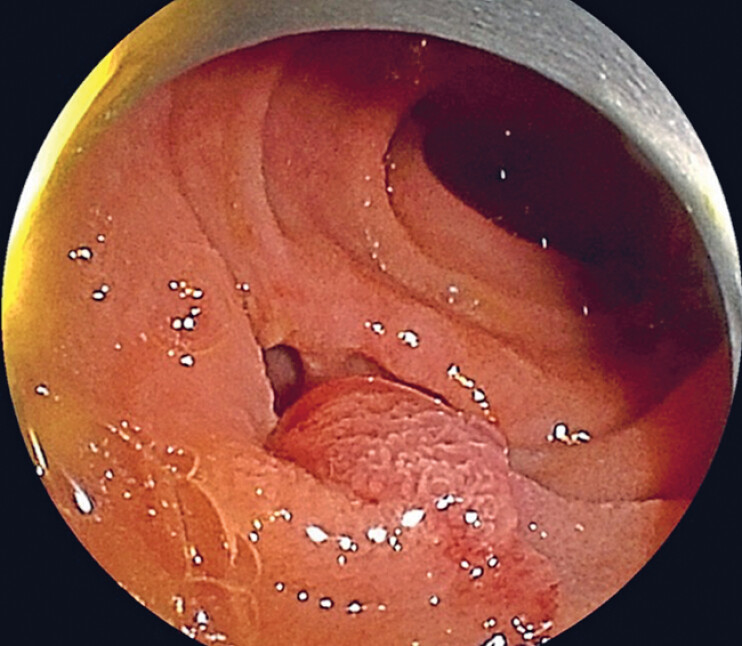
Double-balloon endoscopy showing the papilla located in the diverticulum in a 76-year-old man with a gastrectomy and Roux-en-Y reconstruction and a common bile duct stone.

Biliary cannulation of a peridiverticular papilla achieved using the multiloop traction method in a patient with Roux-en-Y anatomy. EPBLD, endoscopic papillary large-balloon dilation; M-loop, multiloop; PAD periampullary diverticulum.Video 1

A SureClip (Micro-Tech, Nanjing, China) attached to a multiloop thread was used to grasp the duodenal mucosa near the papilla ( Fig. 2 ). Additional clips were hooked to the free loop and attached to the oral side of the intestinal wall. This provided a traction force that pulled the papilla out of the diverticulum. The bile duct opening was visualized and biliary cannulation was achieved ( Fig. 3 , Fig. 4 ). Finally, endoscopic large-balloon dilation was performed, and the bile duct stone was successfully removed. The patient was discharged 4 days after the procedure without complications.

**Fig. 2:**
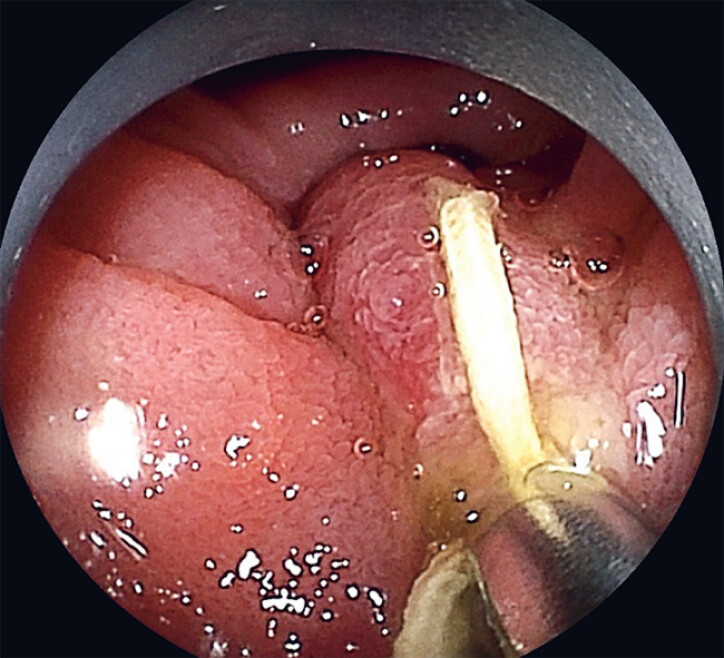
An endoscopic clip was deployed to grasp the duodenal mucosa near the papilla.

**Fig. 3:**
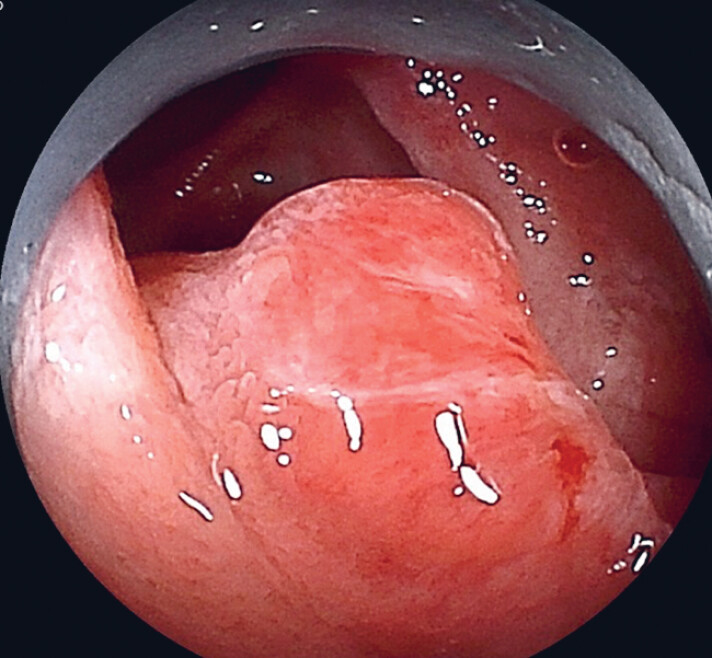
The multiloop traction provided an adequate view for biliary cannulation.

**Fig. 4:**
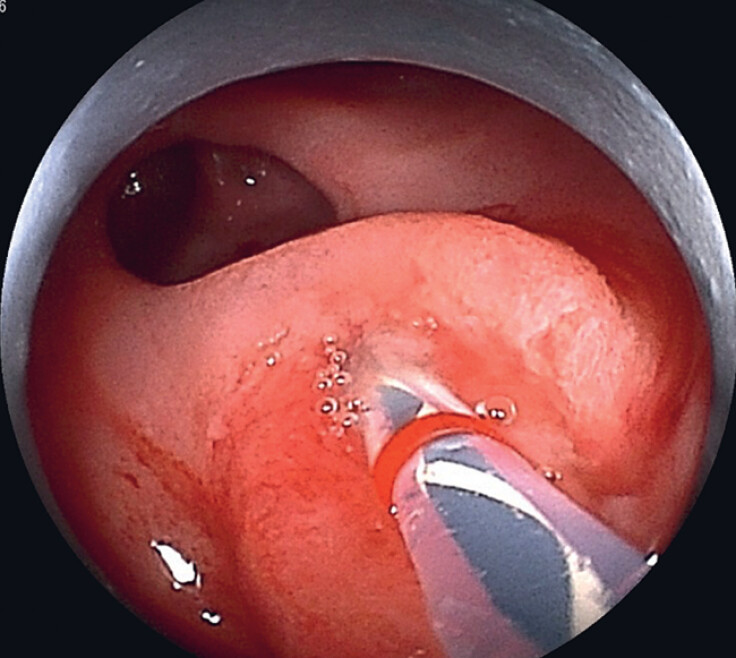
Biliary cannulation was achieved.

Biliary cannulation in a surgically altered anatomy is often challenging because of the instability of the endoscope and the limitations of the devices used in balloon-assisted endoscopy. Some case reports have described that the multiloop method provides a sufficient view of the papilla for biliary cannulation in normal anatomy. However, its use in patients with surgically altered anatomy has not been reported to date. This case suggests that the multiloop method is also useful for biliary cannulation in cases of periampullary diverticula with post-surgical altered anatomy.

Endoscopy_UCTN_Code_TTT_1AR_2AB

## Notes

Endoscopy E-Videos https://eref.thieme.de/e-videos
*E-Videos* is an open access online section of the journal *Endoscopy* , reporting on interesting cases and new techniques in gastroenterological endoscopy. All papers include a high-quality video and are published with a Creative Commons CC-BY license. Endoscopy E-Videos qualify for HINARI discounts and waivers and eligibility is automatically checked during the submission process. We grant 100% waivers to articles whose corresponding authors are based in Group A countries and 50% waivers to those who are based in Group B countries as classified by Research4Life (see: https://www.research4life.org/access/eligibility/ ). This section has its own submission website at https://mc.manuscriptcentral.com/e-videos .
